# Interatrial conduction time is associated with left atrial low voltage area and predicts the recurrence after single atrial fibrillation ablation

**DOI:** 10.1002/joa3.12820

**Published:** 2023-01-23

**Authors:** Kei Hirota, Akira Fukui, Takanori Yamaguchi, Masaki Takahashi, Hidekazu Kondo, Hidefumi Akioka, Tetsuji Shinohara, Kunio Yufu, Koichi Node, Naohiko Takahashi

**Affiliations:** ^1^ Department of Cardiology and Clinical Examination Oita University Yufu Japan; ^2^ Department of Cardiovascular Medicine Saga University Saga Japan

**Keywords:** ablation, atrial fibrillation, intra‐atrial conduction time, left atrial low voltage area

## Abstract

**Background:**

Interatrial conduction time (IACT) prolongs in fibrotic left atrium. We tested the hypothesis that IACT is related to left atrial low voltage area (LVA) and predicts the recurrence after single atrial fibrillation (AF) ablation.

**Methods:**

One hundred sixty‐four consecutive AF patients (79 non‐paroxysmal) who underwent initial ablation in our institute were analyzed. IACT and LVA were defined as interval from the onset of P‐wave to the basal left atrial appendage (P‐LAA) activation, and area with bipolar electrogram < 0.5 mV covering over 5% of the total left atrial surface area during sinus rhythm, respectively. Pulmonary vein antrum isolation, non‐PV foci ablation, and atrial tachycardia (AT) ablation were performed without substrate modification.

**Results:**

LVA was frequently identified in patients with prolonged P‐LAA ≥ 84 ms (*n*  = 28) compared with patients with P‐LAA < 84 ms (*n*  = 136). Patients with P‐LAA ≥ 84 ms were older (71 ± 10 vs. 65 ± 10 years, *p*  = .0061), and had more frequent non‐paroxysmal AF (75% vs. 43%, *p*  = .0018), larger left atrial diameter (43.5 ± 4.5 vs. 39.3 ± 5.7 mm, *p*  = .0003), and higher E/e’ ratio (14.4 ± 6.5 vs. 10.5 ± 3.7, *p*  < .0001) compared with P‐LAA < 84 ms patients. After a mean follow‐up period of 665 ± 153 days, Kaplan–Meier curve analysis showed that AF/AT recurrences was more frequently observed in patients with prolonged P‐LAA (Log‐rank *p*  = .0001). Additionally, univariate analysis revealed that P‐LAA prolongation (OR = 1.055 per 1 ms, 95% CI: 1.028–1.087, *p*  < .0001) and the existence of LVA (OR = 5.000, 95% CI: 1.653–14.485 *p*  = .0053) were predictors of AF/AT recurrences after single AF ablation.

**Conclusions:**

Our results suggested that prolonged IACT as measured by P‐LAA was associated with LVA and predicts AT/AF recurrence after single AF ablation.

## 1 INTRODUCTION

Animal studies have shown that interatrial conduction time (IACT) is prolonged in the fibrotic left atrium (LA) and plays an important role in the maintenance of atrial fibrillation (AF).^1^, ^2^, ^3^, ^4^ We have experimentally demonstrated that LA fibrosis, induced either by continuous infusion of angiotensin II or high‐fat diet, were associated with IACT prolongation, which was defined as time of interelectrode activation between the right atrium and left atrium in mice.^2^, ^3^, ^4^ Additionally, greater probability of atrial fibrillation (AF) was provoked by electrical stimulation in these models. From these studies, we concluded that conduction disturbance expressed as IACT prolongation was mainly caused by interstitial LA fibrosis.^2^, ^3^, ^4^

Left atrial low voltage area (LVA) detected by bipolar voltage mapping during sinus rhythm is considered as a marker of atrial fibrotic tissue.^5^, ^6^ LVA is also an independent predictor of AF/atrial tachycardia (AT) recurrence after AF ablation.^5^, ^6^, ^7^ Indeed, we recently reported that patients with LVA had more frequent recurrence of AT/AT after pulmonary vein antrum isolation (PVAI) compared with those without LVA.^7^ In addition, Miyamoto et al showed that local conduction slowing was observed at the LVA.^8^ However, the relationship between IACT and LVA and the impact of IACT prolongation on AF/AT recurrence after AF ablation are unknown.

Therefore, we tested the hypothesis that (1) IACT, defined as P‐wave‐left atrial appendage conduction time (P‐LAA) recorded during sinus rhythm, relates with LVA and that (2) prolongation of P‐LAA predicts the AF/AT recurrence after single AF ablation procedure.

## 2 METHODS

### 2.1 Study population

A total of 164 consecutive AF patients who underwent initial catheter ablation from July 2017 to April 2019 in Oita University hospital were included. Paroxysmal (PAF), persistent (PeAF), and long‐standing persistent AF (LS‐PeAF) were defined according to the HRS/EHRA/ECAS consensus reports.^9^ A written informed consent was obtained from all patients, and the study was approved by the committee on human research at Oita University.

### 2.2 Electrophysiological study and catheter ablation of AF

Transesophageal echocardiograms and/or contrast computed tomography scans were performed before the ablation to exclude the presence of LA thrombi. Anti‐arrhythmic drugs were discontinued for at least 5 half‐lives before the ablation procedure except for amiodarone.

Procedural preparations were performed as described previously.^10^ Ablation procedures were performed in the postabsorptive state with deep sedation using propofol and fentanyl. A duo‐decapolar catheter was placed in the coronary sinus via the right jugular vein and a temperature monitoring probe was inserted in the esophagus to prevent esophageal injury.

LA geometry and voltage map were obtained using EnSite NavX™ 3D mapping system (Abbott Medical). Bipolar voltage mapping of the LA was performed during sinus rhythm using a 20‐pole circular mapping catheter with a 1‐mm electrode length and 2‐mm interelectrode spacing (Reflexion HD™; Abbott Medical). For patients with AF rhythm, cardioversion was performed to restore sinus rhythm. LVA was defined as the area with a bipolar peak‐to‐peak voltage amplitude < 0.5 mV and covering over 5% of the total LA surface area. The total LVA area was calculated as the percentage of LA surface area excluding the PV antral region, left atrial appendage (LAA) orifice, and mitral valve. P‐LAA was defined as interval from the earliest P‐wave onset to the basal LAA activation and total atrial activation time was defined as interval from the earliest P‐wave onset to the latest LA activation site during sinus rhythm, with the exception of one patient with sinus arrest, who was recorded during right atrial pacing. P‐wave duration was measured in II or V1 lead during sinus rhythm. Pulmonary vein antrum isolation (PVAI) was performed using a contact sensing catheter (TactiCath™; Abbott Medical) through steerable sheath (Agilis™; Abbott Medical). Radiofrequency energy was applied in a dragging fashion with the temperature and power limited to 40 °C and 30 W, respectively. The endpoint of PVAI was completion of entrance and exit block from the PVs. Intravenous Isoproterenol (6–10 μg) injection and burst pacing from the distal site of the coronary sinus electrode catheter was performed to induce atrial tachycardia (AT) and AF from non‐PV foci. When a AT and/or non‐PV focus was identified, additional ablation was performed at the focus. Empiric superior vena cava (SVC) isolation and cavotricuspid isthmus (CTI) linear ablation was added at the discretion of the attending physician. No additional substrate modification was performed.

### 2.3 Follow‐up of atrial tachyarrhythmia

A 12‐lead electrocardiogram was obtained at each visit for at least 1, 3, 6 months, and every 6 months thereafter up to 24 months. In addition, 24‐h Holter monitoring was performed 3, 6, 12, and 24 months after the procedure. A 3‐month blanking period was introduced after the ablation. Any AF/AT lasting ≥ 30 s was considered as a recurrence.

### 2.4 Statistical analysis

Statistical analysis was performed using the JMP software, version 11.0 (SAS). Normally distributed data are expressed as mean ± standard deviation and non‐normally distributed data as median and interquartile range. Univariate analyses of various clinical variables were performed using unpaired t‐tests for normally distributed data, and Wilcoxon's rank sum test for non‐normally distributed data. Fisher's exact test was used for categorical data.

## 3 RESULTS

### 3.1 Relationship between P‐LAA and LVA

**FIGURE 1:**
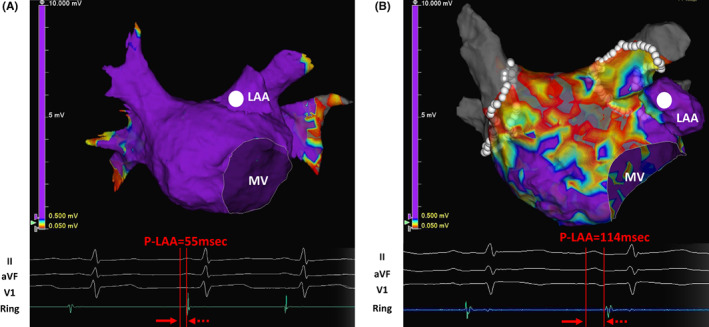
Measurement of the P‐wave‐left atrial appendage (LAA) conduction time (P‐LAA) using the surface electrocardiogram leads (II, aVF, and V1) and intracardiac electrograms detected by the EnSite NavX™ 3D mapping system. P‐LAA was defined as the interval from P‐wave onset in lead II to the basal LAA activation during sinus rhythm. (A) P‐LAA in patient without left atrial low voltage area (LVA) and (B) P‐LAA in a patient with LVA.

**FIGURE 2:**
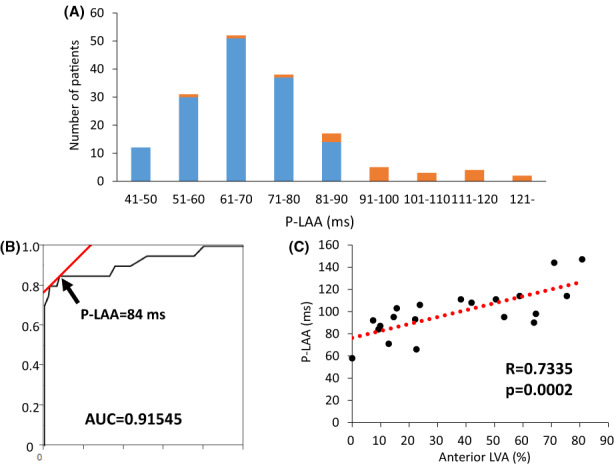
Relationship of P‐wave‐left atrial appendage (LAA) conduction time (P‐LAA) and the existence of left atrial low voltage area (LVA). (A) Distribution of P‐LAA in patients with LVA (orange) and without LVA (blue). (B) ROC curve for predicting LVA. P‐LAA ≥ 84 ms was the cutoff point for predicting LVA in the present cohort. (C) Relation between P‐LAA and anterior LVA.

Figure 1 shows the representative cases with normal P‐LAA without LVA (Figure 1A) and with prolonged P‐LAA with LVA (Figure 1B). The patient shown in Figure 1A had P‐LAA of 55 ms without LVA. In contrast, the patient shown in Figure 1B had prolonged P‐LAA of 114 ms and convincing LVA. Figure 2A shows the relationship between P‐LAA and the prevalence of LVA. Patients with prolonged P‐LAA ≥ 84 ms (*n* = 28) were compared with patients with P‐LAA < 84 ms (*n* = 134) to investigate whether P‐LAA predicts LVA by generating a ROC curve (Figure 2B). LVA was more frequently detected in patients with prolonged P‐LAA ≥ 84 ms than in patients with P‐LAA < 84 ms (2% vs. 55%, *p*  < .0001). Relation between P‐LAA and percentage of anterior LVA is shown in Figure 2C. P‐LAA was closely related to anterior LVA (R = .7335, *p*  = .002).

### 3.2 Characteristics of LVA

LVA was detected in 20 of the 164 patients included in the study. The mean extent of LVA was 24 ± 17% of the total LA surface area. The distribution of LVA was 19 (95%), 16 (80%), 12 (60%), 10 (50%), 8 (40%), 1 (5%), 0 (0%) in the anterior, septum, roof, posterior, inferior, lateral, and LAA, repetitively.

### 3.3 Patient characteristics

Table 1 shows the characteristics of the 164 enrolled patients. Eighty‐five patients had PAF, 50 had PeAF, and 29 had LS‐PeAF. LA thrombi were not detected in any of the 164 patients. Patients with P‐LAA ≥ 84 ms were older (71 ± 10 vs. 65 ± 10, *p*  = .0061), had more frequently nonparoxysmal AF (75% vs. 43%, *p*  = .0018), had higher level of serum NT pro‐BNP (540 [484–1072] vs. 212 [79–542] pg/ml, *p*  = .0007), had greater LA diameter (43.5 ± 4.5 vs. 39.3 ± 5.7 mm, *p* = .0003), and had a higher E/e’ ratio (14.4 ± 6.5 vs. 10.5 ± 3.7, *p* < .0001) compared with P‐LAA < 84 ms patients.

**TABLE 1:** Patient characteristics

	P‐LAA > 84 ms (*n* = 28)	P‐LAA≤84 ms (*n* = 136)	*p*‐value
Nonparoxysmal AF, *n* (%)	21 (75)	58 (43)	.0018
Age, years old	71 ± 10	65 ± 10	.0061
Female gender, *n* (%)	11 (39)	29 (21)	.0438
BMI, kg/cm^2^	25.8 ± 3.4	24.9 ± 3.6	.2337
Heart failure, *n* (%)	7 (25)	25 (18)	.4210
Hypertension, *n* (%)	22 (78)	75 (55)	.0217
Diabetes mellitus, *n* (%)	8 (29)	17 (13)	.0312
Stroke, *n* (%)	0 (0)	11 (8)	.1192
Vascular disease, n (%)	4 (14)	12 (9)	.3751
Creatinine, mg/dL	0.87 ± 0.21	0.94 ± 0.58	.4903
pro‐BNP, pg/mL	540 (484–1072)	212 (79–542)	.0007
LAD, mm	43.5 ± 4.5	39.3 ± 5.7	.0003
LVDd, mm	46.8 ± 6.8	47.5 ± 5.2	.5430
LVDs, mm	30.7 ± 7.7	31.1 ± 5.5	.7067
LVEF, %	61.3 ± 13.2	62.8 ± 9.4	.4646
E/e', ratio	14.4 ± 6.5	10.5 ± 3.7	<.0001

Abbreviations: AF, atrial fibrillation; BMI, body mass index; BNP, brain natriuretic peptide; LAD, left atrial diameter; LVDd, left ventricular diastolic diameter; LVDs, left ventricular systolic diameter; LVEF, left ventricular ejection fraction.

### 3.4 Catheter ablation of AF

The result of AF ablation is presented in Table 2. The mean P‐LAA were 97.9 ± 16.7 ms and 64.6 ± 9.4 ms in patients with P‐LAA ≥ 84 ms and P‐LAA < 84 ms, respectively (*p* < .0001), and LVA was detected more frequently in patients with P‐LAA ≥ 84 ms than in patients with P‐LAA < 84 ms (17/28 vs. 3/136, *p* < .0001). PVAI was successfully performed in all patients. While SVC isolation was performed more frequently in patients with P‐LAA < 84 ms than in patients with P‐LAA ≥ 84 ms (16/28 vs. 105/136, *p* = .0279), AT ablation was performed more frequently in patients with P‐LAA ≥ 84 ms than in patients with P‐LAA < 84 ms (11/28 vs. 6/136, *p* < .0001). Total procedure time was longer in patients with P‐LAA ≥ 84 ms than in patients with P‐LAA < 84 ms (140 ± 28 vs. 117 ± 28, *p* = .0001). Other parameters including non‐PV foci ablation, CTI ablation, total fluoroscopic time, and total ablation time did not differ between the two groups.

**TABLE 2:** Catheter ablation data

	P‐LAA > 84 ms (*n* = 28)	P‐LAA≤84 ms (*n* = 136)	*p*‐value
P‐LAA, ms	97.9 ± 16.7	64.6 ± 9.4	<.0001
LVA, *n* (%)	17 (61)	3 (2)	<.0001
Pulmonary vein antrum isolation, *n* (%)	28 (100)	136 (100)	1.0000
Superior vena cava isolation, *n* (%)	16 (57)	105 (77)	.0279
Cavotricuspid isthmus ablation, *n* (%)	26 (93)	123 (90)	.6863
Atrial tachycardia ablation, *n* (%)	11 (39)	6 (4)	<.0001
non‐PV foci ablation, *n* (%)	0 (0)	5 (4)	.3028
Total procedure time, min	140 ± 28	117 ± 28	.0001
Total fluoroscopic time, min	10.6 ± 5.0	9.2 ± 4.2	.1168
Total ablation time, s	2022 ± 560	1851 ± 399	.0575

Abbreviations: LVA, left atrial low voltage area; P‐LAA, P‐wave‐left atrial appendage conduction time.

### 3.5 Ablation outcome and predictor of AF/AT recurrence

**FIGURE 3:**
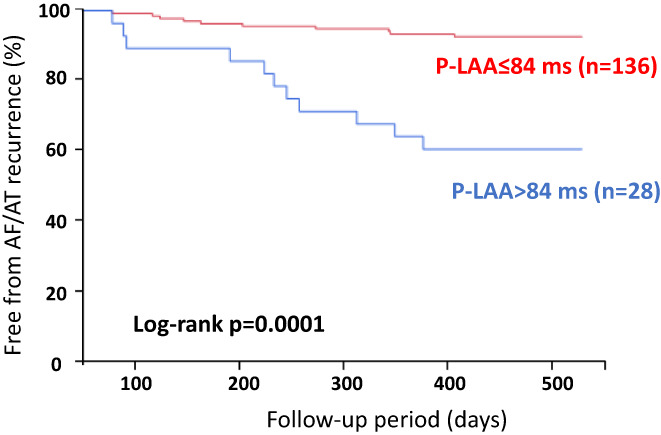
Kaplan–Meier curve for AT/AF recurrence after single AF ablation. Recurrence rate was significantly higher in patients with P‐LAA ≥ 84 ms compared with patients with P‐LAA < 84 ms (Log‐rank *p* = .0001).

Findings on freedom from AF/AT recurrence after single session in patients with P‐LAA ≥ 84 ms and P‐LAA < 84 ms are shown in Figure 3. After a mean follow‐up period of 665 ± 153 days, Kaplan–Meier curve analysis revealed that AF/ AT recurrences were observed more frequently in patients with P‐LAA ≥ 84 ms than in those with P‐LAA < 84 ms (Log‐rank *p* = .0001). In addition, univariate analysis showed that P‐LAA and LVA were predictors of AF/AT recurrence (Table 3). Further multivariate analysis was not preformed because of the multicollinearity between P‐LAA and LVA. On the other hand, LAD and nonparoxysmal AF did not predict AF/AT recurrences in the present study.

**TABLE 3:** Predictors of AT/AF recurrence

	Univariate analysis		
	Odds ratio	95% CI	*p*‐value
P‐LAA, per 1 ms	1.055	1.028–1.087	<.0001
LVA	5.000	1.653–14.485	.0053
Age, per 1 year old	0.989	0.949–1.035	.6174
Nonparoxysmal AF	1.512	0.603–3.918	.3805
Hypertension	2.449	0.904–7.816	.0968
pro‐BNP, per pg/ml	1.000	0.999–1.000	.9665
LAD, per 1 mm	1.022	0.943–1.112	.6033
E/e’, per 1 ratio	1.021	0.919–1.117	.6647

Abbreviations: AF, atrial fibrillation; AT, atrial tachycardia; BNP, brain natriuretic peptide; LAD, left atrial diameter; LVA, left atrial low voltage area; P‐LAA, P‐wave‐left atrial appendage conduction time.

### 3.6 Relationship between parameters of left atrial conduction and LVA

Table 4 shows the relationship between parameters of left atrial conduction and LVA including P‐LAA, P‐wave duration, and total atrial activation time. Univariate analysis showed that all three parameters were predictors of LVA. After multivariate analysis, P‐LAA was the only predictor of LVA. Multivariate analysis was not performed between P‐LAA and total atrial activation time because of the multicollinearity of the two parameters. Measurement of total atrial activation time revealed that the latest activation site of LA was LAA in 9 (5%) and lateral wall in 155 (95%) patients.

**TABLE 4:** Predictors of LVA

	Univariate analysis			Multivariate analysis		
	Odds ratio	95% CI	*p*‐value	Odds ratio	95% CI	*p*‐value
P‐LAA, per 1 ms	1.185	1.105–1.229	<.0001	1.062	1.022–1.112	.0006
P‐wave duration, per 1 ms	1.028	1.002–1.055	.0340	1.024	0.992–1.061	.1567
Total atrial activation time, per 1 ms	1.119	1.070–1.106	<.0001			

Abbreviations: LVA, left atrial low voltage area; P‐LAA, P‐wave‐left atrial appendage conduction time.

## 4 DISCUSSION

The main findings of the present study were as follows, (1) prolonged P‐LAA (longer than the cutoff point of 84 ms) was associated with LVA, (2) patients with prolonged P‐LAA ≥ 84 ms had more frequent AF/AT recurrence after single procedure of AF ablation compared with those with P‐LAA < 84 ms, and (3) univariate analysis including type of AF, LAD, and presence of LVA revealed that existence of LVA and prolonged P‐LAA ≥ 84 ms were the two predictors of AF/AT recurrence. To our knowledge, this is the first study to reveal the relationship between P‐LAA and LVA. Our study also demonstrated for the first time that, prolonged P‐LAA is a predictor of AT/AF recurrence after single AF ablation procedure.

Although direct evidence in a large cohort is lacking, two technologies are recommended to identify atrial fibrosis in human LA. One technique is delayed enhancement magnetic resonance imaging (DE‐MRI) and the another is LVA detected by bipolar voltage mapping during sinus rhythm.^5^, ^6^ McGann et al reported that surgical biopsies of LA endocardial wall demonstrated that tissue fibrosis matched with regions of DE‐MRI, promising the relation of DE‐MRI and atrial fibrosis.^5^ In addition, the same group showed that there was a qualitative correlation between regions of DE‐MRI and LVA in small cohort of AF patients.^6^ In the present study, prolonged P‐LAA was associated with LVA, indicating that advanced LA fibrosis might be responsible for the prolongation of P‐LAA.

Both DE‐MRI and LVA have been reported to be independent predictors of AF/AT recurrence after AF ablation. Marrouche et al showed that atrial fibrosis estimated by DE‐MRI was independently associated with likelihood of recurrent arrhythmia in the multicenter, prospective, observational cohort DECAAF study.^11^ We and Rolf et al reported that LVA was an independent predictor of recurrence of AF/AT after PVAI in patients with AF, and that additional LVA‐based substrate modification would improve the outcome.^12^, ^13^ Moreover, patients with prolonged P‐LAA ( ≥ 84 ms) experienced more frequent AT/AF recurrence compared with P‐LAA < 84 ms patients when analyzing data by ROC curve using the cutoff point of 84 ms. Together, it is likely that patients with prolonged P‐LAA have advanced atrial fibrosis, which may be the cause of grater AF/AT recurrence. Additionally, previous study by Miyamoto K et al showed that LVA revealed significant conduction slowing.^8^ Consistent with this result, in the present study, all patients with prolonged P‐LAA had anterior wall LVA and P‐LAA was closely related to percentage of anterior LVA, which indicates that LVA is the main source of P‐LAA prolongation. In our current study, P‐LAA was a better predictor of LVA compared with P‐wave duration. On the other hand, total atrial activation time could be a substitute for P‐LAA.

Univariate analysis revealed that existence of LVA and P‐LAA were the two predictors of AF/AT recurrence. However, measuring P‐LAA is simpler compared with creating full voltage map of the left atrium. To create an accurate voltage map, the physician must be well‐trained to attach the mapping catheter adequately to the LA wall with sufficient contact. In addition, when the activation wavefront moves perpendicular to the mapping electrode pair, there will be minimal to no difference in the unipolar signals, resulting in a low bipolar electrogram.^14^ Compared with voltage mapping, measurement of P‐LAA does not require neither skilled technique nor specific equipment to assess atrial fibrosis.

## 5 LIMITATIONS

There are several limitations to this study. First, this was neither a randomized nor multicenter study and the number of patients was not large enough to reach a definite conclusion. Second, the follow‐up methodology of AF recurrence was not strict, and it is possible that some asymptomatic recurrences were missed.

## 6 CONCLUSION

In conclusion, P‐LAA was related to LVA and was an independent predictor of AT/AF recurrence after single AF ablation.

## CONFLICT OF INTEREST STATEMENT

All authors have no conflict of interest related to the present study.

## ETHICS APPROVAL STATEMENT

The study was approved by the committee on human research at Oita University (No. 1283, 9/15/2017).

## PATIENT CONSENT STATEMENT

Not applicable

## CLINICAL TRIAL REGISTRATION

Not applicable.
